# A Case of Refractory Peptic Ulcer with Choledochoduodenal Fistula Successfully Treated by Biliary Stent Placement Using an Ultrathin Endoscopic Rendezvous Technique: A Case Report

**DOI:** 10.1002/deo2.70338

**Published:** 2026-04-21

**Authors:** Yuki Miyashita, Akinobu Koiwai, Eri Urita, Masazumi Takemoto, Takehito Itoh, Nana Inomata, Morihisa Hirota, Kennichi Satoh

**Affiliations:** ^1^ Division of Gastroenterology Tohoku Medical and Pharmaceutical University Miyagi Japan

**Keywords:** biliary drainage, choledochoduodenal fistula, ERCP, rendezvous technique, ultrathin endoscopy

## Abstract

Choledochoduodenal fistula (CDF) is an abnormal communication between the common bile duct and the duodenum. While gallstones are the primary cause, peptic ulcer‐related CDF has become rare due to the widespread antiulcer therapy. Nevertheless, CDF remains clinically relevant as symptoms are often vague and diagnosis is frequently incidental. We report the case of a 79‐year‐old woman with vomiting, epigastric pain, and back pain due to a refractory duodenal ulcer complicated by CDF. Endoscopic retrograde cholangiopancreatography (ERCP) using a standard duodenoscope was attempted to facilitate fistula closure by biliary stent placement; however, scope passage was prevented by severe duodenal stenosis. Therefore, ERCP was successfully performed using an ultrathin endoscope with a rendezvous technique. A guidewire was advanced through the CDF into the biliary tract and retrieved at the major papilla. Subsequently, a 5‐Fr double‐pigtail plastic stent was placed in the left intrahepatic bile duct. This approach achieved effective biliary drainage, promoting ulcer healing and complete fistula closure without surgery. The patient was discharged without complications. This case highlights the clinical utility of the ultrathin endoscope‐assisted rendezvous ERCP as a minimally invasive option for CDF with duodenal obstruction when conventional ERCP is not feasible.

## 1 Introduction

Choledochoduodenal fistula (CDF) is an uncommon complication of peptic ulcer disease (PUD). Because clinical findings are often nonspecific, CDF is usually discovered incidentally during radiologic or endoscopic evaluation. There is no consensus regarding optimal management, which ranges from conservative medical therapy to surgical repair. Previous reports have suggested that diversion of bile flow combined with antiulcer therapy may promote fistula closure [1]. Here, we report a rare case of refractory duodenal ulcer complicated by CDF that was successfully treated with biliary stent placement using an ultrathin endoscopic rendezvous technique, resulting in complete fistula closure and ulcer healing.

## 2 Case Report

**FIGURE 1:**
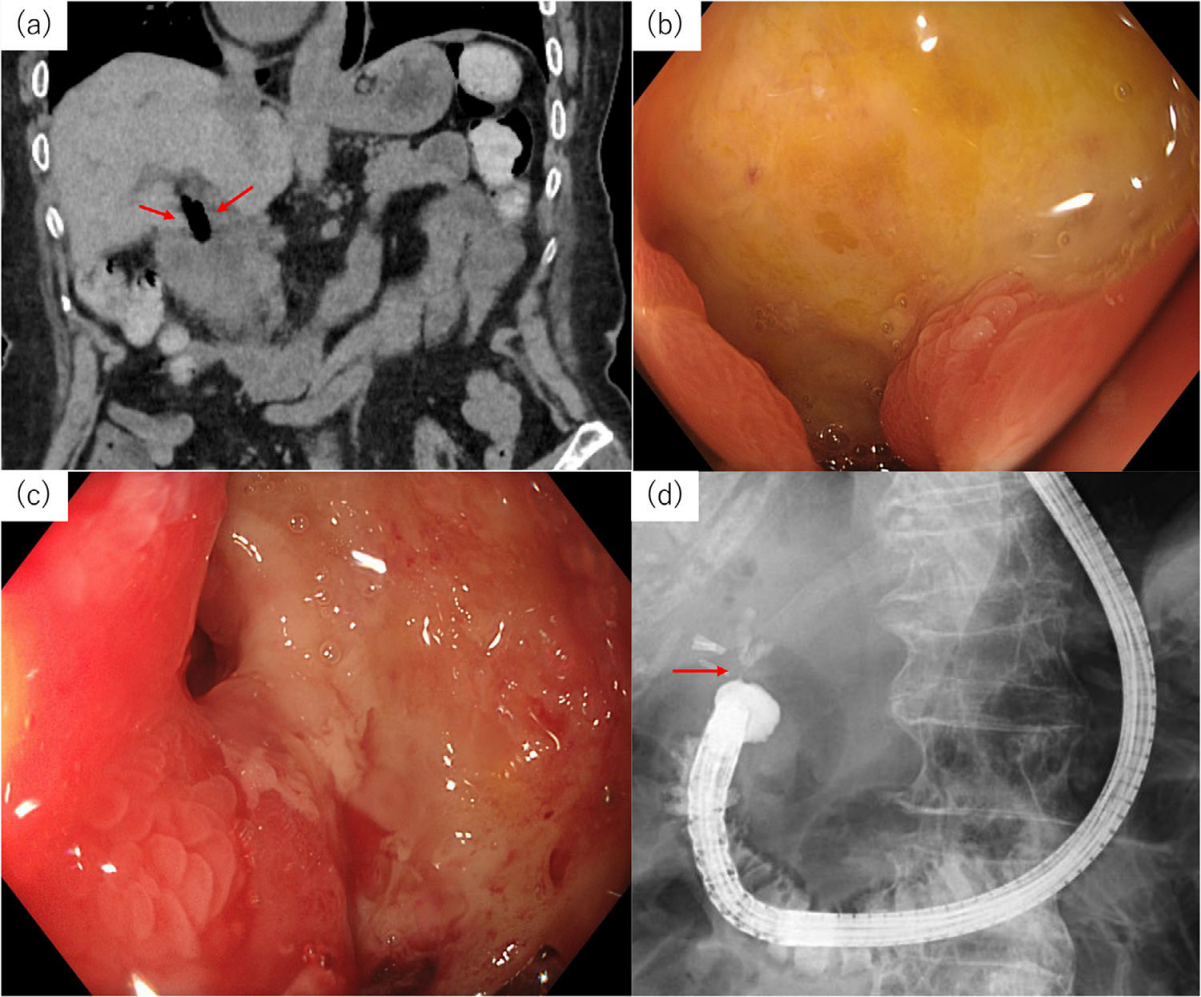
(a) Non‐contrast CT demonstrated the presence of air within the common bile duct (red arrows). (b, c) Endoscopic image showing an ulcerative lesion with a small fistula in the duodenal bulb. (d) Under fluoroscopic guidance, the endoscope was applied to the fistula, and a contrast study demonstrated leakage of a contrast medium into the common bile duct (red arrow). CT, computed tomography.

**FIGURE 2:**
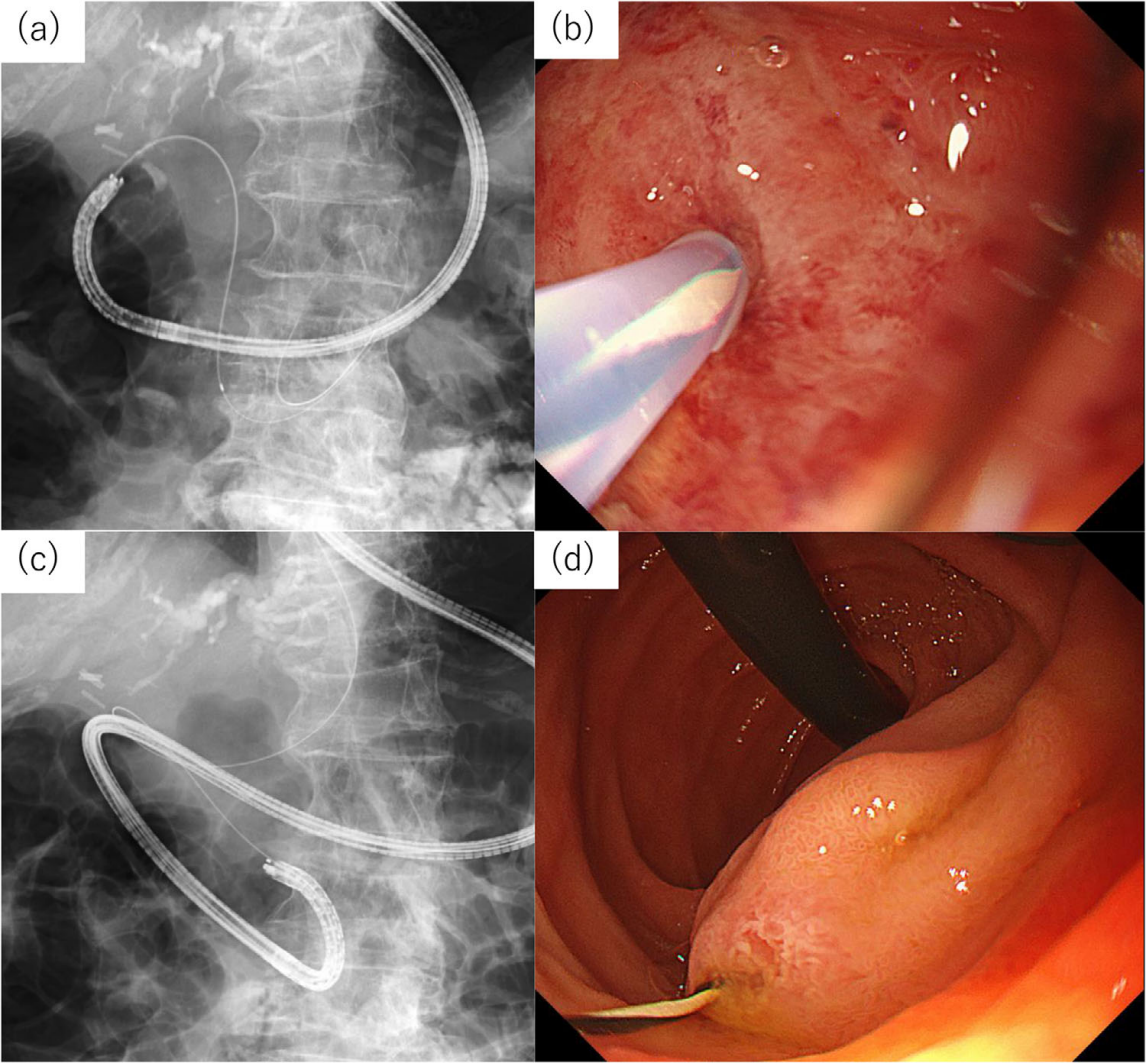
(a, b) The guidewire was advanced through the fistula into the duodenal lumen via the papilla. (c, d) The ultrathin endoscope was reinserted and retroflexed in the descending portion of the duodenum, and the guidewire was retrieved with biopsy forceps.

**FIGURE 3:**
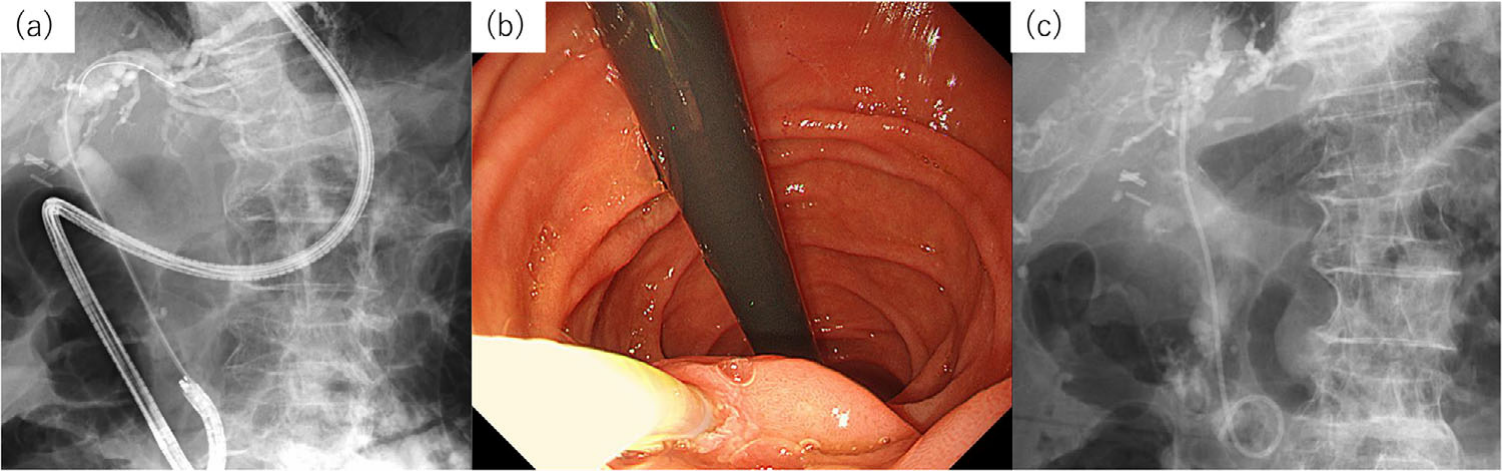
(a) Under fluoroscopic guidance, cannulation of the left intrahepatic bile ducts was carried out. (b) Endoscopic image during stent insertion. (c) Fluoroscopic view confirming successful simultaneous insertion of the double pigtail plastic stent.

**FIGURE 4:**
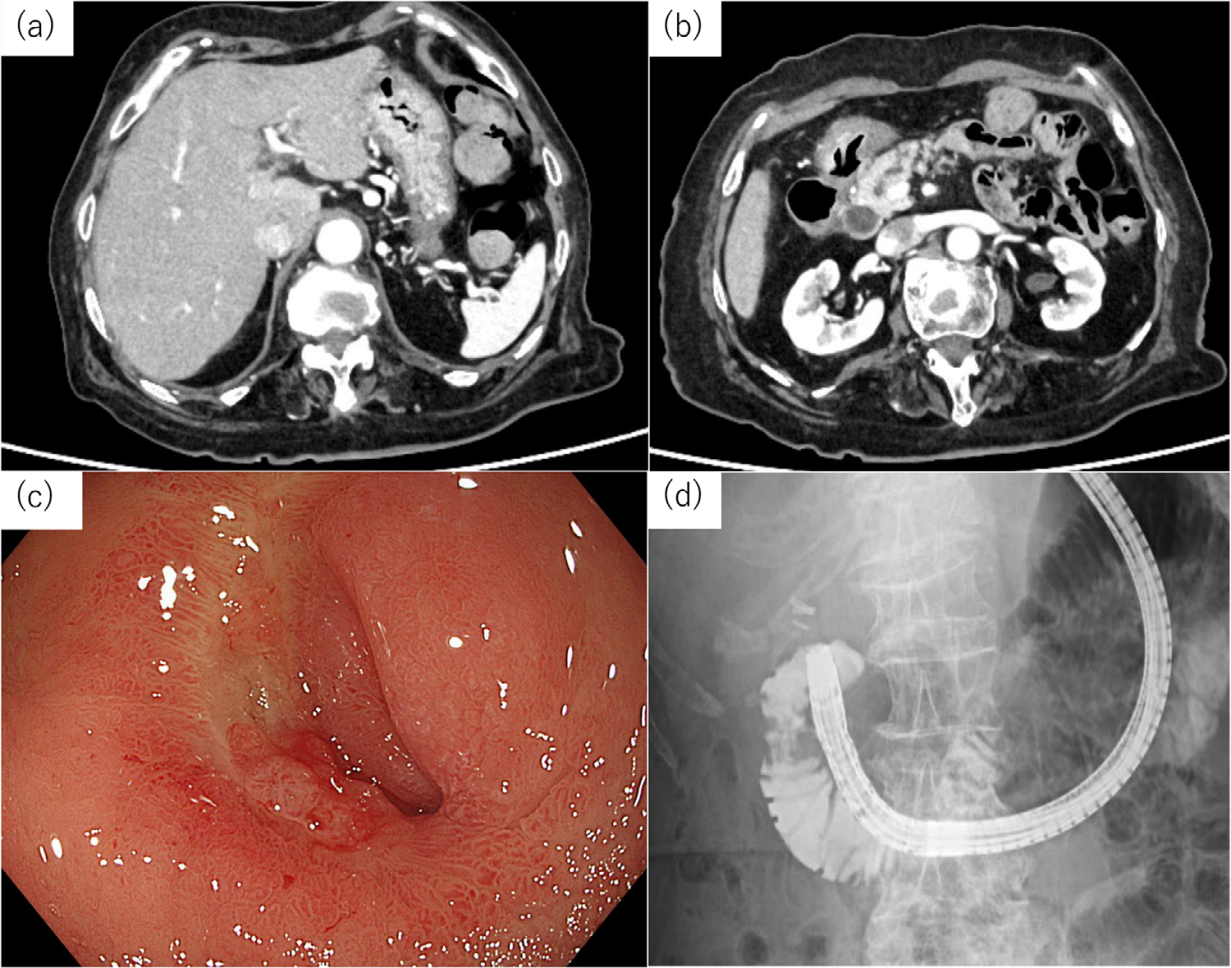
(a, b) CECT demonstrated resolution of pneumobilia and improvement of the intestinal wall thickening. (c) EGD revealed scarring of the ulcer and closure of the fistula. (d) Barium contrast was administered into the duodenal bulb, but no entry into the bile duct was observed. CECT, contrast‐enhanced Computed Tomography; EGD, esophagogastroduodenoscopy.

A 79‐year‐old woman presented with vomiting, epigastric pain, and back pain. She had undergone a cholecystectomy 14 years earlier. Two weeks before admission, she had been prescribed tramadol, acetaminophen, and non‐steroidal anti‐inflammatory drugs (NSAIDs) for lumbar pain without concomitant gastroprotective therapy such as a proton pump inhibitor (PPI). Physical examination revealed conjunctival pallor and tenderness in the epigastrium without fever. Laboratory tests showed leukocytosis (10,100/µL), anemia (hemoglobin 7.2 g/dL), and mildly increased cholestatic enzymes. Non‐contrast computed tomography (CT) revealed wall thickening extending from the pylorus to the duodenal bulb, fat stranding around the pancreatic head, and pneumobilia within the common bile duct (CBD) and intrahepatic ducts (Figure 1a). Esophagogastroduodenoscopy (EGD) demonstrated reflux esophagitis and a large duodenal ulcer (Figure 1b); biopsy showed chronic active inflammation without malignancy. After fluid resuscitation, renal function improved, enabling contrast‐enhanced CT, which showed persistent pyloroduodenal wall thickening and increased pneumobilia. These findings raised suspicion for CDF. Repeat EGD identified a fistulous opening in the ulcer base (Figure 1c). Under fluoroscopic guidance, contrast injection through the opening confirmed communication between the duodenum and the CBD (Figure 1d). NSAIDs were discontinued on admission, and pain control was managed with acetaminophen while PPI therapy was continued. However, persistent pneumobilia and inflammatory changes suggested ongoing bile flow through the fistula, and endoscopic biliary diversion was planned to reduce bile exposure to the ulcer and facilitate healing. ERCP using a standard duodenoscope (TJF‐Q290V; Olympus, Tokyo, Japan) was attempted; however, the procedure failed because severe duodenal stenosis due to chronic ulcer‐induced deformity prevented scope passage. Therefore, ERCP was performed using an ultrathin endoscope (GIF‐1200N; Olympus; outer diameter, 5.4 mm), in conjunction with the rendezvous technique. Because the lumen was markedly narrowed, the small‐caliber endoscope enabled safe access to the fistula. A 0.025 guidewire (EndoSelector; Boston Scientific, Marlborough, MA, USA) was advanced through the CDF into the biliary tract and manipulated to exit into the duodenum via the ampulla. An ERCP catheter

(StarTip V; Olympus) was inserted into the biliary tract (Figure 2a,b). After confirming guidewire passage into the duodenum, the endoscope was withdrawn while leaving the guidewire in place (Figure 2c). The ultrathin endoscope was reinserted into the duodenum alongside the guidewire (Figure 2d). The guidewire emerging from the ampulla was s grasped by a biopsy forceps (Figure 3a), and retrieved through the accessory channel. To prevent friction‐related mucosal injury to the pharynx and esophagus, an ERCP catheter was advanced over the oral side of the guidewire as a protective sheath. Deep biliary cannulation was achieved using an ERCP catheter over the guidewire placed through the CDF (Figure 3b). The initial guidewire was subsequently removed, and another 0.025‐inch guidewire (Fielder 25; ASAHI INTECC, Aichi, Japan) was inserted into the biliary system through the ERCP catheter (Figure 3c,d). A 5‐Fr double‐pigtail plastic stent was subsequently placed in the left intrahepatic bile duct. The key procedural steps are shown in Video S1. On day 15 after stent placement, EGD confirmed improvement of the duodenal ulcer and closure of the CDF, and the stent was subsequently removed. After 3days of removing the stent in the bile duct, CT showed improvement in the thickening from the gastric pylorus to the duodenal bulb (Figure 4a,b). At the same time as EGD, contrast injection was performed, confirming the disappearance of the previously observed opacification of the bile duct and the closure of the CDF (Figure 4c,d). Because the duodenal stricture was secondary to scarring and ulcer scarring, sequential endoscopic balloon dilations were performed, which successfully relieved the stenosis. Endoscopic sphincterotomy after improvement of the duodenal stenosis was considered to reduce intraductal pressure. However, because the fistula had already closed completely and the patient remained asymptomatic, we decided against sphincterotomy in view of the risk of procedure‐related adverse events, particularly post‐ERCP pancreatitis, and opted for careful follow‐up. No recurrence of CDF or duodenal ulcer has been observed for 10 months after stent removal.

## 3 Discussion

CDF secondary to PUD is a rare entity caused by ulcer penetration into the CBD. In the modern PPI era, its incidence has markedly declined; however, sporadic cases still occur, particularly in association with NSAID‐induced ulceration or *Helicobacter pylori* infection [1, 2]. Among the etiologies of spontaneous biliary‐enteric fistulas, gallstone‐related inflammation is the most common, whereas penetrating peptic ulcers are less common [3]. Other reported causes include malignancy, Crohn's disease, previous biliary surgery, endoscopic sphincterotomy, blunt abdominal trauma, and inflammatory conditions involving the peribiliary region [4].

In the present patient, continuous NSAID use without gastroprotection likely contributed to ulcer formation and subsequent fistulization. The decline in peptic ulcer‐related fistulas over recent decades is largely attributable to advances in ulcer therapy, including PPI treatment and eradication of *H. pylori* [5]. In our patient, the bile duct appeared relatively dilated on CT and fluoroscopy. While mild ductal dilation can be seen after cholecystectomy, bile stasis and increased intraductal pressure might also have contributed to the persistence of bile flow through the suprapapillary fistula. Transpapillary stenting may have reduced intraductal pressure and preferentially diverted bile flow toward the papilla, thereby promoting fistula closure.

Clinical manifestations of CDF are variable. Although fever, abdominal pain, and jaundice may occur, many patients are asymptomatic, and diagnosis is often suggested by pneumobilia on imaging [6]. Ulcer‐related CDF may be detected incidentally during imaging or endoscopy performed for nonspecific symptoms, and awareness of this entity is important to avoid misinterpretation as gastrointestinal perforation [1, 7]. In this case, the absence of cholangitis and jaundice delayed recognition, and careful correlation between CT findings and endoscopy was essential.

There is no standardized treatment algorithm for CDF associated with PUD, and management ranges from medical therapy to surgery depending on symptoms and complications [1]. Antiulcer therapy with PPIs is generally first‐line. However, when bile flow through the fistula persists, biliary diversion may be required to promote healing of both the ulcer and the fistula [1, 8]. Conservative management with PPIs and antibiotics is often adequate for small or asymptomatic fistulas; nevertheless, persistent bile leakage can delay ulcer healing, and several reports have described successful outcomes using biliary stenting to divert bile flow [9, 10]. In our case, stent placement rapidly reduced inflammation and resulted in fistula closure.

This case demonstrates that a rendezvous technique using an ultrathin endoscope can overcome anatomical limitations−such as duodenal deformity and fixed stenosis−that preclude passage of a standard duodenoscope. The small‐caliber endoscope can traverse a narrowed lumen more readily while potentially reducing the risk of perforation. Several reports have described biliary drainage using rendezvous techniques via PTBD or EUS guidance, as well as drainage using ultrathin endoscopes. However, to the best of our knowledge, no published reports have described ultrathin endoscope‐assisted rendezvous biliary drainage performed through a CDF. This approach may be a useful therapeutic option for CDF complicated by gastrointestinal stenosis and may be particularly beneficial in elderly or frail patients for whom surgery carries a high risk.

This report describes a single case; therefore, generalizability is limited. Patient selection is crucial, as this approach requires: (i) an identifiable fistulous opening allowing stable catheterization, (ii) the ability to traverse the stenotic segment with an ultrathin scope, and (iii) successful antegrade guidewire passage through the distal bile duct and papilla. Potential risks and failure modes include perforation in an inflamed/stenotic duodenum, cholangitis, guidewire‐induced injury, and inability to achieve stable scope positioning (including the need for retroflexion/inversion depending on anatomy). In addition, the small working channel of an ultrathin endoscope restricts accessory choice and stent caliber, which may limit applicability.

In conclusion, ultrathin endoscope‐assisted rendezvous ERCP was especially useful when a standard duodenoscope could not traverse a stenotic duodenum. This minimally invasive approach may represent an effective treatment option for refractory peptic ulcer complicated by choledochoduodenal fistula and duodenal obstruction.

## Funding

The authors have nothing to report.

## Conflicts of Interest

The authors declare no conflicts of interest.

## Supporting information




**VIDEO S1**: Ultrathin endoscope‐assisted rendezvous ERCP using the choledochoduodenal fistula. The video shows identification of the fistulous opening, catheter cannulation, antegrade guidewire advancement into the biliary tract, guidewire exit through the major papilla and retrieval (rendezvous), and transpapillary placement of a 5‐Fr double‐pigtail plastic stent.
